# Coping Styles and Quality of Life in Breast Cancer Patients Undergoing Radiotherapy

**DOI:** 10.3390/cancers15235515

**Published:** 2023-11-22

**Authors:** Małgorzata Roszkowska, Katarzyna Białczyk

**Affiliations:** 1 Department of Clinical Neuropsychology, Nicolaus Copernicus University, Collegium Medicum, 85-821 Bydgoszcz, Poland; 2 Department of Public Health, Nicolaus Copernicus University, Collegium Medicum, 85-821 Bydgoszcz, Poland; katarzyna.bialczyk@cm.umk.pl

**Keywords:** quality of life, coping styles, breast cancer, radiotherapy, stress, emotion-focused coping

## Abstract

**Simple Summary:**

Cancer patients often have a hard time coping with their illness and treatment. How they cope can impact their quality of life. This study looked at the coping styles and quality of life of breast cancer patients receiving radiotherapy compared with healthy people. Researchers measured levels of active coping (like making plans and thinking positively), emotion-focused coping (like seeking support), and avoidant coping (like denial). They also assessed mental, physical, and overall quality of life. The results show breast cancer patients engaged less in active coping and had a lower quality of life than healthy controls. In patients, avoidant coping was strongly tied to a worse quality of life in all areas. Active coping was only weakly related to better physical health for patients, though it was strongly linked to mental and overall quality of life in controls. The findings show the heavy burden cancer puts on quality of life. They suggest avoidant coping consistently harms wellbeing. Boosting helpful coping skills may lessen treatment side effects.

**Abstract:**

Purpose: This study examined relationships between coping styles and quality of life (QoL) in oncology patients undergoing radiotherapy compared with healthy controls. Coping styles and QoL were assessed to elucidate connections and inform psychosocial care. Methods: 57 females participated, including 28 breast cancer patients undergoing radiotherapy and 29 healthy controls matched on demographics. Participants completed the COPE Inventory, which measures active, emotion-focused, and avoidant coping levels, and the SF-36, which assesses mental, physical, and overall QoL. Between-group differences were analyzed using the Mann–Whitney U test. Correlations between coping styles and QoL were examined with Spearman’s r. Results: Breast cancer patients showed a significantly lower QoL on all scales versus controls. In patients, active coping weakly correlated with physical QoL, while avoidance moderately to strongly correlated with poorer mental, physical, and overall QoL. In controls, active coping strongly correlated with mental and overall QoL, and avoidance moderately negatively correlated across domains. Conclusion: Maladaptive avoidance coping was strongly related to poorer QoL in both groups, especially patients. Active coping showed limited benefits for patients’ QoL, in contrast with the controls. Those with low scores require effective interventions during radiotherapy, which are crucial for coping with treatment adverse effects.

## 1. Introduction

Quality of life (QoL) has been studied across scientific disciplines and is understood in various ways [1]. WHO defines it as the subjective perception of one’s position in life within a cultural and value system context [2]. Research on this topic provides valuable knowledge on patient functioning and life satisfaction in the context of cancer [3], since modern medicine aims not only to prolong life but also to improve its quality. When analyzing QoL definitions [4], it should be noted that it constitutes a complex evaluation expressing one’s attitude towards life as a whole or specific domains, manifested through cognitive appraisals and emotional assessments [5].

Patient knowledge about radiotherapy is often incomplete, which may increase fear and symptoms of anxiety and depression [6,7]. Numerous studies confirmed associations between QoL indicators and health status, disease characteristics, and treatment methods [8,9,10,11]. The cancer experience is a source of severe stress. Coping style refers to general personality dispositions, manifested in behaviors displayed in threatening situations [12], representing the reactions most often exhibited [13,14].

According to the cognitive transactional concept, stress is defined as a specific relationship between an individual and their environment, which can be appraised as taxing or exceeding resources [15]. It encompasses both environmental demands and individual coping ability. The confrontation occurs through cognitive appraisal—interpreting and evaluating the stressor. This appraisal plays a key role in defining the situation—its demands, threat magnitude, and available resources [16]. Lazarus distinguished two stages of cognitive appraisal: primary, evaluating event significance and threat relevance, and secondary, evaluating coping resources. If an individual judges a transaction as exceeding coping capacity, stress is experienced [17]. Making a secondary appraisal allows them to proceed to the coping stage.

Coping encompasses cognitive and behavioral efforts undertaken in stressful situations, serving two functions—problem solving and emotional regulation [18]. Two independent dimensions are distinguished: information seeking, corresponding to a confrontational style, and information avoidance, representing an avoidant style [19]. This is a fundamental distinction between coping categories. From a cognitive transactional perspective, coping represents constantly changing efforts to deal with specific demands [20]. This process constitutes a response to a specific situation and includes all individual efforts made to cope.

Despite difficult circumstances, some patients find meaning and perceive illness as a turning point, describing life as more valuable and fulfilling, evaluating their QoL as better compared to pre-diagnosis [21,22]. Others react oppositely; despite good prognosis and survival chances, they see the diagnosis as a death sentence, reject help, and rate their QoL as low. Regardless of the support and goods present, they see no point in continuing the fight against illness [23].

These differences may be influenced by socioeconomic factors, health/illness perceptions, and values. Lazarus emphasizes the role of interpretation; appraisal shapes situational perception [17]. It plays a key role in defining demands, threat magnitude, and coping resources. Variability is already evident at the illness perception and resource appraisal stages. A situation where an event is appraised as taxing or exceeding resources, threatening wellbeing, is treated as psychological stress [15]. Referring to the conceptualization of relational stress allows for considering chronic illness as a potential stressor, significantly disrupting the subject–environment balance. Patients must adapt to a new, changed situation. Thus, breast cancer patients experience tremendous stress related to health deterioration and life changes. Their reactions to illness may determine their perception of the current situation and QoL [17].

Given the significant psychological burden of cancer, this study aimed to elucidate relationships between oncology patient coping styles and self-reported quality of life.

## 2. Material and Methods

The present study utilized two well-established questionnaires assessing coping styles and quality of life.

The first method applied was the multidimensional COPE Inventory originally developed by Carver et al. [24]. This 60-item questionnaire measures 15 conceptually distinct coping strategies on a 4-point Likert scale [1]. For this study, the validated Polish adaptation of the COPE Inventory was used [25]. The translation to Polish involved back-translation procedures to ensure equivalence. Psychometric analyses were conducted on the Polish version, evidencing good internal consistency and validity [2]. In a review by Kato [25], the original COPE was found to have mean Cronbach’s alphas between 0.62 and 0.85 across coping scales. Test–retest reliability over 2 months ranged from 0.42 to 0.89. The COPE shows evidence of convergent and discriminant validity with related and unrelated constructs. It is considered a reliable and valid measure of coping, assessing a range of cognitive and behavioral coping efforts [25].

Factor analysis of the items indicated that they represent three general coping styles, including (1) active coping, encompassing the strategies of active coping, planning, restraint coping, suppression of competing activities, and positive reinterpretation and growth; (2) seeking social support and focusing on emotions, comprising venting of emotions, use of instrumental social support, use of emotional social support, humor, acceptance, and turning to religion; and (3) avoidance strategies including denial, distraction, ceasing actions, and use of alcohol/drugs [25].

The second tool utilized allowing the measurement of quality of life was the 36-Item Short-Form Health Survey (SF-36) originally developed by John E. Ware, Jr. and Cathy Donald Sherbourne [26]. This is a self-administered multidimensional questionnaire containing 36 statements representing eight domains of quality of life: physical functioning, role limitations due to physical health, bodily pain, general health perceptions, vitality, social functioning, role limitations due to emotional problems, and mental health. These eight subscales can be further aggregated into two composite summary scores indicating overall physical- and mental-health-related quality of life [26]. The validated Polish adaptation of the SF-36 by Tylka and Piotrowicz was applied in the present study [27]. In the Polish version, higher scores denote a lower perceived quality of life, while lower values indicate a better quality of life. The 36 statements have varying response formats ranging from dichotomous (yes/no) choices to 6-point Likert scales. The SF-36 has demonstrated excellent psychometric properties in multiple studies, including high internal consistency reliability, good test–retest reliability, and strong construct validity, showing it to be a sound measure of health-related quality of life [26,27].

An exploratory factor analysis using principal component analysis with Varimax rotation was conducted to reduce the 15 COPE scales into more general coping styles. The analysis extracted 3 main factors explaining 59% of the total variance: an active coping style (29% of variance), a support-seeking/emotion-focused style (19% of variance), and an avoidant style (11% of variance).

The sample consisted of 57 participants—29 healthy female controls (HCs) and 28 breast cancer patients (BCp) after surgery undergoing adjuvant radiotherapy, treated at a single center. All patients underwent radical radiotherapy (RT) to the breast area with a dose of 40.05 Gy in 15 fractions over 3 weeks of treatment. This study was conducted in the second week of radiotherapy.

The HC group consisted of women (mean age 48 years, SD = 9.77). The BCp group consisted of patients (mean age 50 years, SD = 11.14).

All participants gave consent and could refuse to participate or withdraw from the study at any time. The study purpose and procedures were thoroughly explained. Detailed sociodemographic data are presented in Table 1.

## 3. Results

The primary research question concerned QoL and coping differences between groups. QoL was the dependent variable, and coping styles were independent variables. Appropriate analyses were applied, including Spearman’s r correlations and Mann–Whitney U tests. Significant results were denoted with asterisks.

### 3.1. Coping Style Characteristics in BCp and HC

Table 2 presents the mean scores (Ms) and standard deviations (SDs) for the three main coping styles identified in the factor analysis of the COPE questionnaire: the active coping style, support-seeking and emotional-focused style, and avoidant style. Coping styles were operationalized using 15 scales of the COPE questionnaire, which were classified into three general styles. The scores were calculated as arithmetic means of the participants’ responses to individual scales and standard deviation (SD) as a measure of dispersion around the mean.

The data are presented separately for the group of breast cancer patients (BCp) and the healthy control (HC) group.

Oncology patients scored lower on active and support-seeking/emotion-focused coping. The most frequently used strategies were planning, focusing/venting emotions, and seeking instrumental support. Active coping was also common. Alcohol/substance use and humor were the least common. The table shows that compared with healthy controls, breast cancer patients obtained lower scores across all three major coping styles identified in the factor analysis.

**Figure 1:**
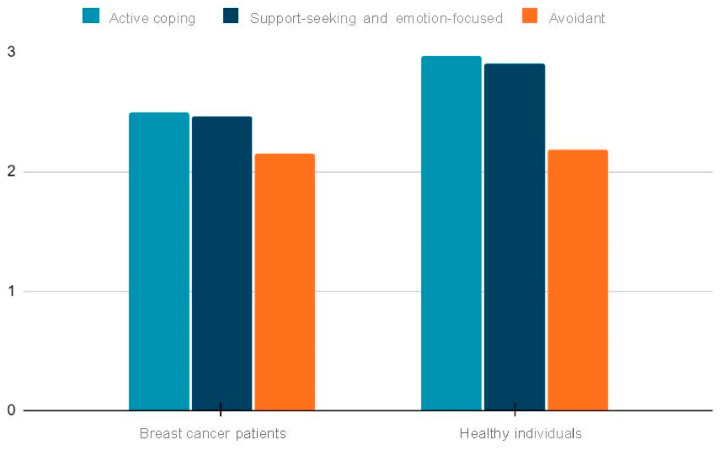
Mean scores for the 3 major coping styles identified in the factor analysis of the COPE questionnaire. Mean scores with standard deviations are presented for active coping, support-seeking/emotion-focused coping, and avoidance coping styles in the breast cancer patient (BCp) and healthy control (HC) groups.

As shown in Figure 1, which displays the three coping styles identified in factor analysis, breast cancer patients scored lower on active and support-seeking/emotion-focused coping.

### 3.2. QoL Characteristics in BCp and HCs

Quality of life was operationalized across three dimensions: overall score, mental health scale, and physical health scale. Table 3 presents the quality-of-life results measured using the SF-36 questionnaire in the two groups studied: breast cancer patients (BCp) and healthy controls (HCs). For each of these dimensions, the table shows the mean (M) and standard deviation (SD) separately for the BCp and HC groups.

In the SF-36 questionnaire, a lower score indicates a worse quality of life.

The obtained data indicate that in each of the analyzed dimensions, breast cancer patients achieved higher average scores than healthy individuals. For example, for the overall quality-of-life score, the mean in the BCp group was −87.63, while in the HC group, it was −53.37. Similar differences were observed for mental health (BCp: −31.26; HC: −21.94) and physical health (BCp: −56.38; HC: −27.13). This indicates a poorer quality of life of breast cancer patients relative to healthy individuals. However, considerable within-group variability was found in both groups, as evidenced by high standard deviation values.

**Figure 2:**
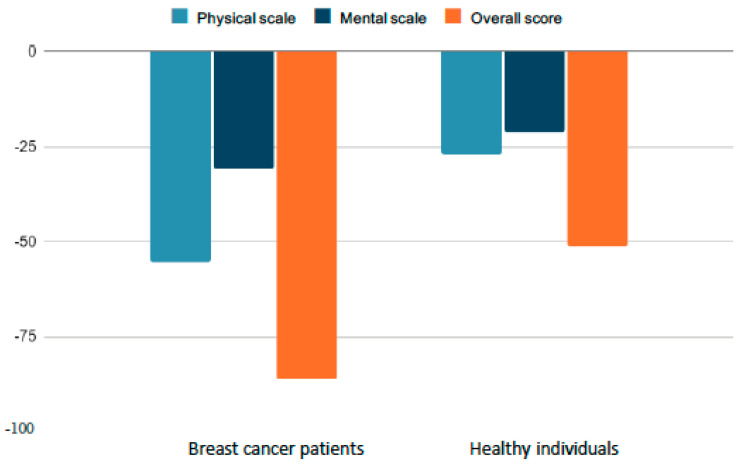
Quality of life evaluation in the studied groups. The figure presents mean scores (Ms) with standard deviations (SDs) for overall quality of life and subscales of physical and mental health in the breast cancer patients (BCp) and healthy control (HC) groups.

Analysis of between-group differences using Mann–Whitney U test showed statistically significant differences on all scales in Table 4. Average scores are also compared graphically in Figure 2.

Higher scores indicate a better QoL. Oncology patients showed significantly lower QoL on all scales, including total score.

Mann–Whitney U test analysis revealed statistically significant differences in quality of life between the groups on all scales. Healthy controls had significantly higher scores, indicating a better quality of life. The average difference was 7.61 points for overall quality of life (*p* < 0.01), 5.58 points for physical health (*p* < 0.01), and 3.49 points for mental health (*p* < 0.01), as shown in Table 4.

Such considerable decreases in the quality of life for breast cancer patients compared with healthy individuals reflect the multidimensional burden of the disease. These findings underscore the importance of regularly monitoring quality of life to identify patients requiring additional support.

### 3.3. Correlations between Coping Styles and QoL

Table 5 presents Spearman’s correlation coefficients between the three main coping styles (active coping, seeking support and emotional focus, avoidance) and the three quality of life dimensions (overall quality of life, mental health, physical health) separately for the group of breast cancer patients (BCp) and the control group of healthy individuals (HC). Besides the correlation coefficients r, *p* values corrected using the Bonferroni method are provided.

Among oncology patients, active coping did not significantly correlate with mental or overall QoL after Bonferroni correction. A weak positive correlation (r = 0.39) was found with physical QoL; however, it was not statistically significant (*p* = 0.117).

In contrast, a moderate negative correlation (r = −0.43) was found between mental health QoL and avoidant coping in oncology patients, though it did not reach significance after correction (*p* = 0.086). Stronger negative correlations were seen between avoidant coping and physical health (r = −0.72, *p* = 0.001) and total QoL score (r = −0.64, *p* = 0.002), which remained significant after Bonferroni adjustment. This highlights robust associations between avoidance coping and worse QoL across domains among breast cancer patients.

In the healthy control group, active coping showed a very strong positive correlation (r = 0.55, *p* = 0.001) with mental health QoL after correction. A moderate positive correlation (r = 0.41) with overall QoL score was not significant after adjusting for multiple tests (*p* = 0.123). Active coping was not significantly related to physical health in controls.

Avoidant coping negatively correlated with mental, physical, and total QoL in controls; however, these associations were not significant after Bonferroni adjustment (all *p* > 0.086).

The findings show some similarities but also differences regarding active and avoidant coping associations with quality of life between groups. Avoidant coping was strongly related to worse QoL in patients, while active coping benefits were specific to mental QoL in healthy controls.

## 4. Discussion

The present study makes an important contribution to understanding coping dynamics and quality of life in breast cancer patients undergoing radiotherapy. The findings provide insights into the complex psychological and emotional adjustment challenges posed by chronic illness. In particular, the limited benefits of typically advantageous active coping strategies for breast cancer patients highlight the unique coping demands of this population [28,29]. Active coping strongly supports quality of life for healthy individuals, as seen in the control group.

Compas et al. [30] similarly found weaker effects of active coping on emotional distress in breast cancer patients compared with healthy women. They suggested age-related differences in appraisal and coping resources may play a role. However, oncology patients’ associations were diminished between active coping and mental or overall quality of life and only weak for physical quality of life [14]. Xiao C. et al. [31], in their study of 39 breast cancer patients undergoing radiotherapy, reported that the overall quality of life did not change significantly during or after radiotherapy. However, a higher BMI was associated with a worse quality of life in terms of physical functions.

The present study builds on this by showing decreased active coping effects on quality of life, especially during radiotherapy. These findings also build on Hack and Degner’s [32] longitudinal results linking avoidant coping with poorer psychological adjustment over time in breast cancer patients. The present cross-sectional data further emphasize the risks of avoidance coping for oncology patients’ wellbeing and functioning. Based on accumulated evidence, avoidance appears to be consistently maladaptive across cancer types and stages [12].

The coping profile of newly diagnosed patients using planning, emotional expression, and seeking instrumental support aligns with past studies [13]. Active engagement is common early post-diagnosis, while avoidance increases over time for some [33]. This stage may represent a critical window for fostering adaptive coping skills before avoidant patterns become entrenched.

Finally, reduced quality of life across domains in breast cancer patients versus controls reflects the multifaceted burden identified in previous quality of life research [2,3]. However, subjective quality of life does not always align with objective factors [34], underscoring the complex role of appraisal and values. Avoidant coping appears to be especially harmful in the cancer context, undermining long-term wellbeing. This aligns with prior research linking avoidance with poorer mental health outcomes in chronic illness [35].

Additionally, the concerning quality-of-life gap between patients and controls reflects the heavy multidimensional burden of cancer [9]. Routine screening is crucial for identifying issues impacting individual functioning [3]. Other authors’ research shows interventions during treatment can improve coping and quality of life [12].

Cognitive impairments and sexual dysfunctions can also be important in impacting the quality of life of both younger and older breast cancer patients [36]. Younger and older patients exhibit different characteristics of these disorders and employ different coping strategies. Every breast cancer patient should be supported throughout their entire oncological history in order to increase quality of life and compliance with treatment [36].

Study limitations provide direction for future research. The cross-sectional design captures coping and quality of life at one point. Longitudinal data could provide richer insights into coping trajectory throughout diagnosis and treatment stages [12]. The small sample also limits generalizability. Larger, diverse samples could better represent the heterogeneous cancer population [1].

Qualitative designs could also better elucidate coping process nuances. Interviews or focus groups may shed light on specific strategies and resources patients find helpful across illness stages [22]. Mixed-methods approaches combining surveys and interviews could provide a comprehensive understanding of coping facilitators and barriers [37].

Overall, the findings underscore the need for multifaceted psychosocial care across the cancer continuum to meet coping demands and improve functioning [38]. With a deeper understanding of coping dynamics, interventions could be tailored to maximize quality of life [15].

## 5. Conclusions

This study elucidates important connections between coping styles and quality of life in breast cancer patients undergoing radiotherapy. Maladaptive avoidance coping strongly negatively correlated with mental, physical, and overall quality of life. Conversely, typically beneficial active coping showed limited advantages, only weakly relating to physical health.

Oncology patients frequently used planning, emotional expression, and instrumental support seeking, representing active engagement. However, they showed a substantially lower quality of life compared with healthy controls, indicating a heavy multidimensional cancer burden.

Assessment enables identifying patients struggling with poorer quality of life who require additional support. Targeted interventions during radiotherapy may improve coping skills and offset treatment side effects. Fostering adaptive coping is crucial for enhancing psychological adjustment and maximizing wellbeing in cancer.

Further research should explore facilitators and barriers in coping processes through qualitative designs. Comprehensive understanding can inform tailored psychosocial care to meet the unique demands posed by chronic illness.

## Figures and Tables

**Table 1:** Sociodemographic data in studied groups.

Variable	Specification	*n*		%	
		BCp *n* = 28	HCs *n* = 29	BCp	HCs
Age	30–40	4	6	14.3	20.7
	41–60	16	17	57.1	58.6
	61–70	8	6	28.6	20.7
Place of residence	City < 50 k	0	2	0	6.9
	City 50–100 k	14	14	50	48.3
	City > 100 k	14	13	50	44.8
Education	Tertiary	4	7	14.2	24.2
	Vocational	12	13	42.9	44.8
	Secondary	12	9	42.9	31.0
	Primary	0	0	0	0
Marital status	Married	11	12	39.3	41.4
	Informal Relationship	16	17	57.1	58.6
	Single	1	0	3.6	0

*n*—number of patients, BCp—breast cancer patients, HCs—healthy controls.

**Table 2:** Mean and standard deviation of coping styles.

	M ± SD			
Coping Styles	BCp		HC	
active coping	2.34	±0.64	2.82	±0.65
planning	2.64	±0.63	3.11	±0.57
seeking instrumental social support	2.67	±0.63	2.96	±0.71
seeking emotional social support	2.24	±0.76	2.85	±0.78
avoidance of competing activities	2.35	±0.71	2.86	±0.58
turning to religion	2.35	±1.02	2.89	±0.77
positive re-evaluation and development	2.37	±0.92	2.89	±0.74
refraining from action	2.38	±0.66	2.98	±0.72
acceptance	2.43	±0.72	2.76	±0.69
focusing on emotions and their discharge	2.67	±0.75	2.94	±0.69
denial	2.35	±0.91	2.13	±0.85
distraction	2.42	±0.78	2.22	±0.81
cessation of action	2.32	±0.75	2.18	±0.98
use of alcohol or other psychoactive substances	1.58	±0.76	1.77	±0.69
use of humor	1.85	±0.74	2.09	±0.86

M—mean use, SD—standard deviation in use, BCp—breast cancer patients, HC—healthy control.

**Table 3:** Coping style usage (Mean ± SD).

QoL	M ± SD			
	BCp		HC	
Mental scale	−31.26	±13.57	−21.94	±13.24
Physical scale	−56.38	±20.14	−27.13	±22.63
Overall score	−87.63	±31.44	−53.37	±26.43

**Table 4:** Significance of differences in the assessed quality of life (mean ± SD).

QoL	Significance of Differences between the Study Groups	Standard Error Difference
Mental scale	*p* < 0.01	±3.49
Physical scale	*p* < 0.01	±5.58
Overall score	*p* < 0.01	±7.61

**Table 5:** Correlations between coping styles and quality of life in both studied groups.

QoL	Active Coping		Seeking Support and Emotional Focus		Avoidance Styles	
	BCp	HC	BCp	HC	BCp	HC
Mental scale	0.16 *p* = 1.000	0.53 *p* = 0.001	0.23 *p* = 1.000	0.07 *p* = 1.000	−0.43 *p* = 0.086	−0.43 *p* = 0.086
Physical scale	0.39 *p* = 0.117	0.41 *p* = 0.123	0.25 *p* = 1.000	0.11 *p* = 1.000	−0.72 *p* = 0.001	−0.40 *p* = 0.123
Overall score	0.33 *p* = 0.495	0.55 *p* = 0.001	0.26 *p* = 1.000	0.07 *p* = 1.000	−0.64 *p* = 0.002	−0.4 *p* = 0.123

## Data Availability

The datasets used and/or analyzed during the current study are available from the corresponding author on reasonable request.
